# Combining Chemistry and Engineering for Hepatocellular Carcinoma: Nano-Scale and Smaller Therapies

**DOI:** 10.3390/pharmaceutics12121243

**Published:** 2020-12-20

**Authors:** Danielle L. Stolley, Anna Colleen Crouch, Aliçan Özkan, Erin H. Seeley, Elizabeth M. Whitley, Marissa Nichole Rylander, Erik N. K. Cressman

**Affiliations:** 1 Department of Biomedical Engineering, The University of Texas, Austin, TX 78712, USA; Danielle.stolley@austin.utexas.edu (D.L.S.); mnr@austin.utexas.edu (M.N.R.); 2 Interventional Radiology, M.D. Anderson Cancer Center, Houston, TX 77030, USA; accrouch@mdanderson.org (A.C.C.); emwhitley@mdanderson.org (E.M.W.); 3 Wyss Institute for Biologically Inspired Engineering at Harvard University, Boston, MA 02115, USA; Alican.Ozkan@wyss.harvard.edu; 4 Department of Chemistry, University of Texas at Austin, Austin, TX 78712, USA; erin.seeley@utexas.edu

**Keywords:** hepatocellular carcinoma, cirrhosis, hyperthermia, ablation, transarterial chemoembolization, microfluidics, mass spectrometry imaging

## Abstract

Primary liver cancer, or hepatocellular carcinoma (HCC), is a major worldwide cause of death from carcinoma. Most patients are not candidates for surgery and medical therapies, including new immunotherapies, have not shown major improvements since the modest benefit seen with the introduction of sorafenib over a decade ago. Locoregional therapies for intermediate stage disease are not curative but provide some benefit. However, upon close scrutiny, there is still residual disease in most cases. We review the current status for treatment of intermediate stage disease, summarize the literature on correlative histopathology, and discuss emerging methods at micro-, nano-, and pico-scales to improve therapy. These include transarterial hyperthermia methods and thermoembolization, along with microfluidics model systems and new applications of mass spectrometry imaging for label-free analysis of pharmacokinetics and pharmacodynamics.

## 1. Introduction

Primary liver cancer, or hepatocellular carcinoma (HCC), is the sixth most prevalent cause of cancer and fourth most common cause of cancer-related mortality globally [1,2]. Within the United States, the predicted mortality from HCC in 2020 is >30,000, making it nearly as frequent as the much more common prostate cancer [3]. Considering that the incidence of HCC is expected to increase in the coming decades, this disease is a significant public health problem. An unfortunate reality confronting patients with HCC is that it most frequently occurs in a setting of underlying cirrhosis that may limit available treatments. The most common causes of cirrhosis have historically been viral hepatitis, alcohol abuse, and dietary aflatoxin exposure. The advent of a vaccine for hepatitis B, screening of donated blood products, and the development of direct-acting antiviral drugs for hepatitis C means that the etiology is shifting away from these conditions as causes for HCC. Alcohol abuse remains a significant cause for HCC, but non-alcoholic fatty liver disease and steatohepatitis (NAFLD/NASH) are becoming much more prominent in the epidemiology of HCC. Screening efforts in at-risk patients primarily take the form of semi-annual ultrasound and serum alpha-fetoprotein level monitoring. Despite the apparently clear logic of this strategy, there is controversy surrounding this approach, as it has not been shown to lead to a decrease in mortality [4]. 

Surgical resection provides a potential cure in patients with well-preserved liver function and localized disease. However, due to factors such as underlying cirrhosis, tumor size, location, or multiplicity of tumors at diagnosis, few patients are candidates for resection [5]. Liver transplant can be an option in some cases, but the demand far exceeds the supply of available donor organs. Taken as a group, the number of patients with HCC receiving a liver transplant is in the range of 5% of those diagnosed. Thus, even without considering costs, transplant is not a realistic option for the majority of patients. 

Medical therapies for HCC were for many years ineffectual. Over a decade ago, sorafenib marked a turning point with a small survival benefit (<3 months in selected patients) [6]. There was much optimism that more and better therapies would soon follow. Between real-world issues with tolerance to sorafenib and little if any improvement from newer drugs such as lenvatinib, regorafenib, or cabozantinib, medical therapy for HCC still faces many challenges [7]. Immune checkpoint inhibitors and combination of other drugs with immunotherapies likewise are being actively studied, and there is much optimism [8,9,10,11]. To date, however, there have been no significant breakthroughs towards a medical cure for HCC. This sets the backdrop for locoregional therapies to treat HCC. In this article, we review various aspects of nano-scale and smaller technologies for treating large or multifocal unresectable HCC. The discussion spans a broad range from patient selection, disease stage, and the technical aspects that affect outcomes to an examination of evidence for success with transarterial chemoembolization (TACE) in intermediate stage patients. We follow this with an assessment of adjunct and emerging transarterial methods at the nano- and smaller scale. We illustrate in some detail new chemistry-based methods and complete the discussion with the coverage of new methods that are well-suited to better model and evaluate these innovative techniques. 

## 2. Patient Selection and Technical Aspects in Large or Multifocal HCC

Globally, HCC patient populations vary in age, degree of cirrhosis, tumor burden at presentation, and underlying etiology. Numerous algorithms have been developed to standardize the patient evaluation process and to stage/stratify patients to various treatment strategies. These classification schemes continually evolve over time to reflect better understanding of liver disease and other developments in therapy [12,13]. The Barcelona Cancer of the Liver Clinic (BCLC) algorithm is perhaps the most widely used scheme, but there are ongoing efforts to further refine patient selection and disposition [14]. Smaller nodules <3 cm are most often treated with radiofrequency or microwave ablation, whereas intermediate stage disease (the focus of this article) is treated with arterial delivery methods. Transcatheter methods lend themselves readily to nano-scale and smaller technologies as discussed further below. TACE arose in Japan and was first reported in the early 1980s [15,16]. Some twenty years later, two randomized clinical trials of TACE established the foundation for future work by demonstrating a significant survival benefit over the best supportive care [17,18]. 

In HCC, new vessels arise from the hepatic artery rather than from the portal vein to support tumor growth. TACE exploits this preferential blood supply from the hepatic artery, allowing targeted delivery of therapy to a tumor. A catheter is advanced under fluoroscopic guidance and therapy is delivered to the tumor’s vascular bed in a highly targeted manner. The goal is complete necrosis of the target tissue with as little collateral damage as possible. This may seem simple enough, but in practice, there are numerous difficulties as outlined below.

From a pharmacological perspective, there are many different drugs currently used in TACE and the procedure itself is also subject to considerable variation [19,20]. Conventional TACE (cTACE) uses ethiodized oil as the basis for an emulsion. However, methods for preparation and deployment of an emulsion are also inconsistent [21]. Likewise, endpoints for the embolization procedure are variable [22]. In response, there are now several drug-eluting beads (DEB-TACE) that aim to provide a standardized dose of a drug, most commonly doxorubicin, with prolonged local release. However, no improvement in overall survival has been shown with added drug compared to embolization alone [23], and there are different compositions for the bead materials and their drug-release profiles [24]. Complicating matters further are differences of opinion on the added value of various cytotoxic and anti-mitotic drugs to the overall outcome [25]. Cells in such an environment may enter a dormant state, and alternative modes for cell survival in such conditions include autophagy, a strategy that recycles cellular components [26]. A case can be made for targeting survival mechanisms under the circumstances rather than blocking cell division, which is already suppressed due to a lack of nutrients and oxygen.

## 3. Measuring Success in TACE for HCC: Evidence from Imaging and Pathology Correlations

While it is widely accepted that TACE is not curative, success is evaluated by imaging criteria from contrast-enhanced computed tomography (CT) or magnetic resonance imaging (MRI) scans. Assessment of the degree of necrosis in treated tumors is made, typically graded as complete response (CR), partial response (PR), stable disease (SD), or progressive disease (PD). The Response evaluation criteria in solid tumors (RECIST) and the newer modified version (mRECIST) [27] have been used for such assessments, as well as the guidelines from the European Association for the Study of the Liver (EASL) [28] and the newer 3D quantitative volumetric variant (qEASL) [29]. Substantial effort in radiomics and artificial intelligence/machine learning is being applied to provide even more accurate assessments of efficacy. However, an important factor to consider in these reports is that very few have direct correlations between imaging and pathology specimens. Of those that do, there is controversy concerning the degree of accuracy [30], but two aspects stand out. First, imaging in most studies tends to overestimate response, with discordance in as many as 20–40% of cases [31,32,33,34]. Conclusions drawn from imaging alone, without some measure of caution, can provide an unwarranted degree of confidence in the effectiveness of treatment, delays in further care, inappropriate selection for transplant, and false hope in patients. Second, TACE, as currently practiced, clearly leaves a significant amount of untreated residual viable tumor in many cases and this occurs more often with larger tumors. When explanted organs are examined in light of the ground truth of histopathology, viable tumor cells have been found in up to 90% of specimens [35,36]. Indeed, compared to the earliest correlative reports, the situation has not substantially improved in 25 years [37]. Adaptations to adverse conditions have been shown to occur in cells that survive TACE, which may further impact overall survival [38,39]. There is, therefore, substantial room for improvement in transarterial therapy.

## 4. Adjunct Strategies: Transcatheter Hyperthermia and Nanotechnologies

In efforts to improve this situation, hyperthermia has been proposed as an adjunct to TACE. We briefly touched on thermal therapy above as it pertains to radiofrequency and microwave ablation, but hyperthermia has relevance in transarterial thermal therapy as well. Device-based methods incorporating hyperthermia have run the spectrum from mild hyperthermia to ablative level temperatures [40]. A detailed account of thermal biology is beyond the scope of this discussion [41]. However, among its many effects, mild hyperthermia is known to incite transient vessel dilation, increase permeability, and it can act as a powerful sensitizer for radiation and chemotherapy [42]. By comparison, ablative level temperatures would be expected to cause vascular damage, coagulation, vessel occlusion, and ischemia. Examples of heated solutions used to deliver heat energy in a locoregional manner include warmed saline and heated ethiodized oil [43,44,45,46,47]. The use of hyperthermia for ablation delivered via catheter seems initially to be an appealing strategy, but it does have limitations. The volume of solution required, the quantity of heat energy borne by heated fluids, heat loss to the surroundings during delivery, and any systemic or whole-body heating must be considered. Practically speaking, it is unlikely that endovascular ablative hyperthermia by itself will be successful strictly based on thermal dose considerations.

An alternative strategy to achieve higher thermal doses in the target tissue while avoiding the problem with the delivery of high volumes is to combine nanotechnology and alternating magnetic fields. This is known as magnetic fluid hyperthermia, and it has been applied as an endoluminal solution (e.g., bladder) through direct interstitial injection into tissue or via catheter in an artery [48,49,50,51]. After the fluid containing magnetic nanoparticles is delivered, an external alternating magnetic field is applied to induce local heating. The interested reader is referred elsewhere as a large body of literature exists concerning specific conditions and composition of nanoparticles [52,53]. 

## 5. Pico-Scale Technology Using Chemical Energy: The Thermoembolic Strategy

A completely different approach towards local hyperthermia is the one that relies on in situ exothermic chemistry as the energy source. The basis of such a strategy is the potential energy within an individual molecule. For scale, this might be considered picotechnology to distinguish it from nanotechnology. The general concept is that a compound is delivered through a catheter and subsequently undergoes a reaction in the tissue to release heat energy. In addition, the product(s) of the chemical reaction can be chosen so as to have additional useful properties. Ischemia will be a natural consequence due to the disruption of the blood supply. With the abrupt onset of multiple extreme changes in the local milieu, efficient yet localized cell death is expected. We call this concept thermoembolization [54].

**Figure 1:**
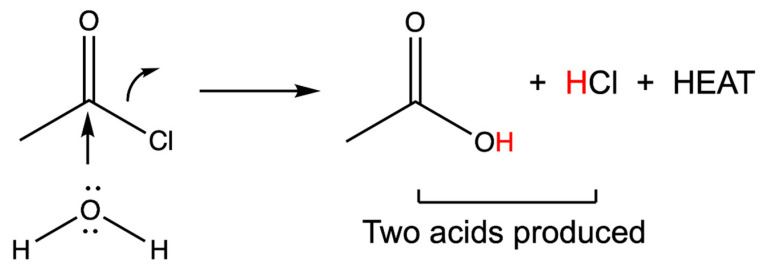
An exothermic reaction as used in thermoembolization. In this example, the electrophile is acetyl chloride, and water serves as the nucleophile. The final products from this case are acetic acid and hydrochloric acid. The red hydrogen atoms are acidic and readily protonate the surrounding tissues. The acid is a lower energy compound than the acid chloride and this difference is manifested as the heat released locally as described further below.

Among several possible categories of exothermic reactions, the hydrolysis of an electrophile is a likely initial choice and is outlined in Figure 1. In this example, the electrophile is acetyl chloride, a readily available and very inexpensive reagent. Compounds such as this are typically used in esterification and amidation reactions with an organic substrate. Here, however, hydrolysis through reaction with the water present in tissues should release a large amount of heat energy, approximately 96 kJ/mole [55,56]. The chloride ion is displaced and a hydrogen ion is released in the process. Thus, for every molecule that reacts, one equivalent of HCl is produced in situ. Furthermore, the final product of the reaction is acetic acid, which even in the absence of added heat energy is a chemical ablation agent that coagulates tissue in its own right [57]. The combination of changes results in rapid vessel thrombosis and thus systemic exposure is extremely limited.

**Figure 2:**
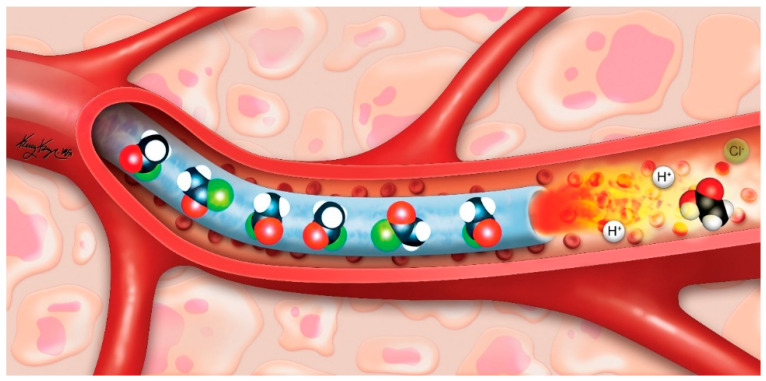
The graphic illustrating the concept for thermoembolization (not to scale). A catheter in the lumen of a blood vessel contains the reactive compound (acetyl chloride, green denotes chlorine atoms) which is delivered using an inert vehicle such as ethiodized oil. On exiting the catheter, reaction occurs leading to local highly denaturing conditions. Conversion of the acid chloride is depicted as downstream presence of acetic acid, which rapidly leads to vessel thrombosis, thus limiting systemic exposure. Reproduced from [54], PLoS ONE, 2018.

Several requirements for transcatheter delivery quickly become apparent for successful application of thermoembolization as a strategy: the use of a single compound (thereby simplifying delivery) that is a non-viscous liquid at room temperature to ensure adequate mixing, that yields no gaseous reaction by-products that could lead to gas embolism, that possesses adequate solubility in a delivery vehicle such as ethiodized oil, and it should have a tractable level of chemical stability, such that special conditions for storage and handling would not be needed. The overall situation is graphically depicted in Figure 2. Here, a microcatheter loaded with acetyl chloride is in the vascular lumen, and as the reagent exits the microcatheter and becomes exposed to the blood and tissues, immediate hydrolysis ensues. By convention, the red atoms represent oxygen atoms and the green atoms represent chlorine atoms. Both hydrochloric and acetic acids are formed in the setting of local heat energy deposition, causing thrombosis and tissue coagulation. Because of the rapid reaction and shutdown of circulation, very little systemic exposure is predicted.

**Figure 3:**
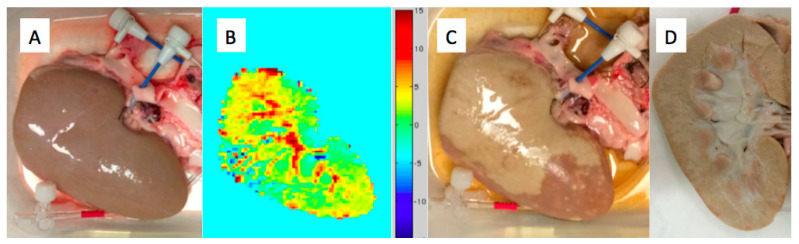
Ex vivo thermoembolization example demonstrating exotherm from the reaction and correlation with the pathology. (**A**) Fresh porcine kidney with a sheath secured in the renal artery for delivery of the reagent solution. (**B**) 5 mm slab MR temperature imaging of the thermal angiogram due to the reaction. (**C**) Gross specimen after reagent delivery showing pale areas of tissue coagulation. (**D**) Bivalve image of the specimen showing near complete internal coagulation.

To be clinically relevant, any exotherm should be of a large enough magnitude to be detected. Thermocouples are often used, but these provide data only from discrete points and could potentially fail to accurately capture the effects if not perfectly positioned. An alternative is to use thermal imaging by the magnetic resonance proton resonance frequency (PRF) shift imaging. This is an established method that captures temperature differences volumetrically by voxel and slice [58]. A proof of concept example is shown in Figure 3, along with the tissue before and after the reaction. In this example, a porcine kidney rather than liver was used for three main reasons. First, the simplified vascular supply greatly facilitated setting up such experiments. Second, PRF shift imaging is a subtraction method and so it is highly sensitive to motion. Ex vivo tissues can be readily stabilized to minimize this issue. Finally, fresh tissue that had been anticoagulated and flushed to purge blood was conveniently available. The reagent was dichloroacetyl chloride (DCACl) in this example, and 2 mL of 4 mol/L solution in ethiodized oil were sufficient to rapidly coagulate the bulk of the parenchyma of the specimen. The PRF image is a coronal slab that is essentially a chemically induced thermal angiogram that maps the energy release from the reaction in tissue. A more detailed and comprehensive investigation of thermal imaging with exothermic chemistry was recently published [59].

**Figure 4:**
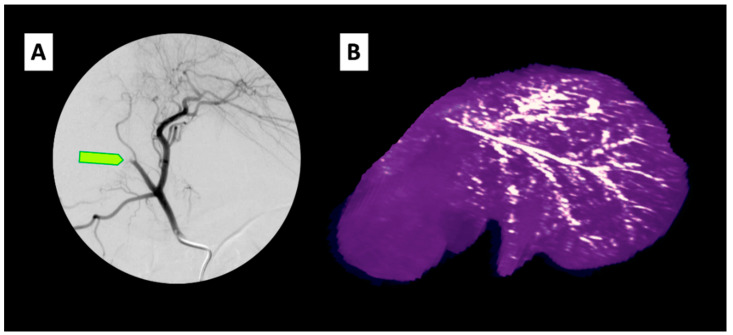
In vivo imaging of thermoembolization in vivo in a pig model. (**A**) Digitally subtracted angiogram following contrast injection through catheter in the common hepatic artery in a pig. Light green arrow indicates abrupt cessation of the flow in the vessel treated using a microcatheter in the target vessel. (**B**) Reconstructed CT image of the liver from the pig obtained the following day showing the branching distribution of the ethiodized oil embolic solution in the arterial tree.

In vivo, delivery via the hepatic artery in a pig model has substantial effects both macroscopically and microscopically [60,61]. Initial results in vivo are encouraging regarding how well the procedure is tolerated [54]. Figure 4 shows a typical digital subtraction angiogram of the common hepatic artery after thermoembolization on the left and a volumetric reformatted CT image of a liver 24 h after the procedure on the right. The yellow arrow indicates the abrupt cut-off of the flow in the treated area, which occurred almost immediately upon delivery. This example shows treatment with approximately 400 µL of a 2 mol/L solution of DCACl in ethiodized oil. The persistent radiopaque arborized embolic material on the scan the following day provides preliminary evidence of a significant level of durability for disruption of blood flow in the target area.

**Figure 5:**
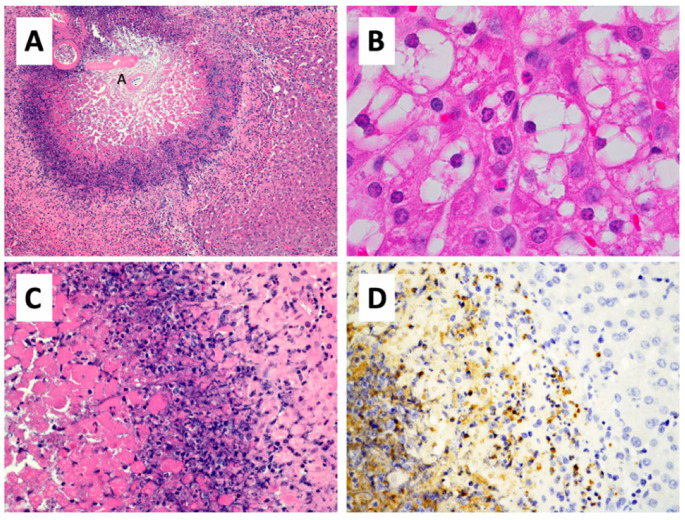
Histopathology of thermoembolization. (**A**) In this section of pig liver that was located downstream from a much larger lesion, thermoembolization results in a targetoid lesion with locally extensive coagulative necrosis that is centered on the hepatic arteriole (annotated as small “A”) and radiates peripherally to encompass portions of several hepatic lobules. Hepatic lobular architecture is preserved centrally, even though constituent cells are necrotic. Zones of lytic necrosis and inflammation are visible as the basophilic zone. In less affected regions of liver (**B**) located more distal to another large thermoembolization lesion, many centrilobular hepatocytes are vacuolated and scattered apoptotic cells are present. (**C**) Non-discrete zones of coagulative necrosis, lysed necrotic hepatocytes, neutrophilic infiltrate, apoptotic cells, and injured but potentially viable hepatic parenchyma are present from left to right in a higher magnification image of a portion of panel A. (**D**) The infiltration of neutrophils is documented by myeloperoxidase immunostaining of a parallel field. Hematoxylin and eosin staining (**A**–**C**) and immunohistochemical staining for myeloperoxidase. Magnification: 100× (**A**), 1000× (**B**), 400× (**C**,**D**).

Histopathology of porcine liver without a tumor that was exposed to exothermic chemical reactions from arterial thermoembolization confirms the development of intense local damage to the arterial wall, as well as to adjacent and downstream lobular structures. Histological changes are typified in photomicrographs (Figure 5), which demonstrate a targetoid lesion centered on a small hepatic arteriole. The damage consists centrally of acute coagulative necrosis surrounded by concentric layers of irreversible cell injury and, more peripherally, zones of reversible cell injury mixed with an early inflammatory cell infiltrate and scattered cells undergoing apoptotic cell death. Much larger areas of necrosis that encompassed and effaced many hepatic lobules were present upstream of this lesion, which is used here to demonstrate the zonal nature of tissue damage from thermoembolization.

Coagulative necrosis of tissues arising from thermal injury has been appreciated for many years [62,63,64]. At sufficient temperatures, the constitutive cells undergo peracute cell death and the architecture of extracellular matrix components is altered. Cell death occurs largely due to transformation of constituent cytoskeletal, enzymatic, and regulatory proteins to a solid state that begins as the temperature reaches 38 °C and progresses to completion at approximately 75 °C. Extensively cross-linked structural collagen proteins undergo denaturation due to disruption of the intramolecular hydrogen bonds between glycine molecules in adjacent chains. Breakdown into smaller peptides does not occur, however, due to the fact that hydrolytic enzymes that would otherwise degrade tissues are also inactivated under these conditions. Depending on the particular exothermic reaction, tissues may also be exposed to a rapid change in pH, which will contribute to further disruption of the secondary and tertiary protein structure and protein–protein interactions. 

In addition to the insults described above, exothermic reactions and subsequent release of contents stored within cell compartments may result in marked osmotic changes within the necrotic and injured/inflammatory zones of the resulting lesion. This hypertonic microenvironment will further disrupt protein function and cell viability [65,66]. In regions with sublethal hypertonic stress alone, cells may employ strategies such as autophagy [67] to re-establish homeostasis, as noted earlier. In combination with other stressors that degrade cell machinery required in this process, survival is less likely.

As noted above in Figure 5, the combined effects of heat, acid, and salt from thermoembolization are that the overall tissue architecture is relatively preserved during the early post-injury period before host repair processes remodel the damaged site. Areas of the liver that undergo thermoembolization have marked coagulative necrosis centered on the hepatic arteriole, radiating from the portal triad and into adjacent hepatic lobules. The shape and arrangement of portal structures are retained and the hepatic cord architecture is somewhat preserved. In the central region, eosin, the pink dye used in routine histopathology that binds to both intact and degraded proteins, demonstrates protein remnants of matrix and dead cells, while hematoxylin staining of DNA and RNA of constituent cells is largely lost. Combined, these features indicate irreversible cell injury and death. More peripherally in the exposure zone where cell damage is less severe, fine cytomorphological features are better preserved.

The histological features described above are consistent with central areas of coagulative necrosis and irreversible cell injury, with a low likelihood of cell survival. At the periphery, furthest from the hepatic arterioles, there is a higher likelihood of reversibility of injury and eventual re-establishment of homeostasis. Ideally, when used to induce necrosis of hepatocellular carcinoma, the zone of frank necrosis or irreversible cell injury would map to the tumor with extension into the surrounding non-neoplastic parenchyma to ensure complete treatment. The extent of damage several hundred microns distant from the arteriole is noteworthy. This encompasses the entire portal triad and considerably beyond. The ability to cause tissue damage extending across multiple hepatic lobules from a single chemoembolization injection approaches that seen with beta-emitters such as ^90^Y in radioembolization without the need for a radioisotope, the high cost, and the logistics associated with half-life and dosimetry.

Beyond the areas of acute coagulative necrosis, sublethal and delayed effects of thermoembolization and host response are evident. Regionally, in less affected areas surrounding the thermoembolization site, many lobules have marked vacuolar degeneration or apoptosis of centrilobular hepatocytes. Based on the available data, we postulate that these findings may be in response to increased local hypoxia due to loss of blood flow and/or “backflow” of reaction products. The amorphous basophilic debris forming one of the outer rings of the targetoid lesion consists of DNA and RNA from ruptured nuclei admixed with intact and degenerating neutrophils. Neutrophils are recruited to the lesion by damage-associated molecular pattern (DAMP) signaling and form an initial host inflammatory and innate immune response.

## 6. Engineering New Micro-Scale Model Systems for the Nano and Pico Scales: Collagen Hydrogels

A challenge in developing safe and effective minimally invasive techniques, such as those described above, is the requirement for models that faithfully mimic vascular, microenvironmental, and metabolic aspects of HCC. Most commonly, animal models have been employed to reflect the complexity of human disease [68]. Rodent models have been used extensively. Pioneering work on embolization was done by Kan et al. over 30 years ago using several animal species [69]. Since then, techniques have been developed for arterial work in rats to mimic embolization in the human hepatic artery, but such work has only been successful in a few highly specialized labs [70,71,72]. Moving up in scale, the rabbit VX2 model has been very frequently employed. However, among the many other issues associated with the model, accurate assessment of necrosis caused by therapy in a model that inherently rather quickly develops extensive spontaneous necrosis dampens many investigators’ enthusiasm. Others have advocated for the woodchuck model [73,74]. This model uses a naturally occurring viral hepatitis that leads eventually to tumors including adenomas and HCC, and it is much closer to human viral disease in that respect. However, numerous disadvantages lead to substantial expense and uncertainty. It requires neonatal inoculation of kits with woodchuck hepatitis virus, continuous husbandry and housing over 2–5 years, managing hibernation cycles and the associated profound metabolic changes, an uncertain time over which tumors develop, and unpredictable statistics arise. Further, unlike the majority of cases of human HCC, there are no fibrotic changes in the liver, and the vessels are small and prone to spasm during procedures. In the future, genetically engineered animals such as the Oncopig [75] and others may find increasing applications for such investigations.

Thus, there is a very real need for alternative technologies that bridge the gap between tissue culture in two dimensions (2D), which provides limited insight into the effects of the microenvironment [76,77] and in vivo studies done with relevant models on a meaningful size scale. Studies have shown that expansion of a 2D tissue culture to a three-dimensional (3D) culture system significantly impacts chemotherapeutic resistance (Özkan et al., n.d.). These studies traditionally include isolated liver bioreactors, liver spheroids, and other static 3D cultures [78,79]. However, most of these models do not represent the cellular and vascular complexity to accurately investigate drug delivery techniques in vitro as they lack the vital context of the hepatic vasculature [80,81,82,83,84]. Formation of functional vasculature in 3D microfluidic devices can be accomplished in several days with or without the influence of flow. It has been previously demonstrated that physiological flow and wall shear stress in an endothelialized vessel can impact the formation of tight junctions and confluency. These, in turn, regulate the permeability of the system to solutes, nutrients, and chemotherapeutics to tumor cells in a 3D microfluidic device [85,86] that are much more representative of in vivo than 2D or static 3D models. Traditionally, these 3D microfluidic models utilize type I collagen derived from rat tails as the primary extracellular matrix (ECM) component for each tissue microenvironment. Collagen concentrations in the ECM of microfluidic platforms can be modulated to replicate healthy and cirrhotic liver stiffness at the time of study to match native liver compression moduli [87]. Environmental regulation of oxygen concentration can be implemented to simulate hypoxic conditions stemming from liver disease or treatment. In addition, ECM matrix composition can be adjusted to incorporate additional ECM proteins such as collagen IV, laminin, and fibronectin or heterogeneous compositions of reconstituted basement membrane proteins (i.e., Matrigel™) to increase the complexity of the microenvironment and provide adhesion molecules and structural functions tailored to the cells and tissues of interest.

We have previously utilized collagen type I for the ECM in vascularized multi-tissue-on-a-chip microfluidic platforms to model breast and liver microenvironments to study the spatiotemporal dynamics of drug transport [86,88]. This shows that the formation of functional vasculature in 3D microfluidic platforms can be accomplished in several days under physiological flow conditions. These models enable us to study the dynamic determination of vessel permeability, the measurement of drug transport, and associated efficacy [85,89]. They also replicate the pharmacodynamics and pharmacokinetics of the human liver when designed according to allometric scaling laws [90]. Moreover, these models provide the basis to investigate cell–cell and cell–matrix interactions in the context of hepatic vasculature [82]. This aspect of the hepatic vasculature in an isolated in vitro model not only permits the visualization of transport and drug efficacy over time, but also allows for further expansion into the study of transarterial therapies in a high-throughput and controllable manner. Furthermore, the ability to expand upon the existing architecture to be more physiologically relevant in the context of the liver sinusoidal structure through the addition of vital cellular components, including stellate cells and Kupffer cells, can permit the study of multicellular interactions. 

**Figure 6:**
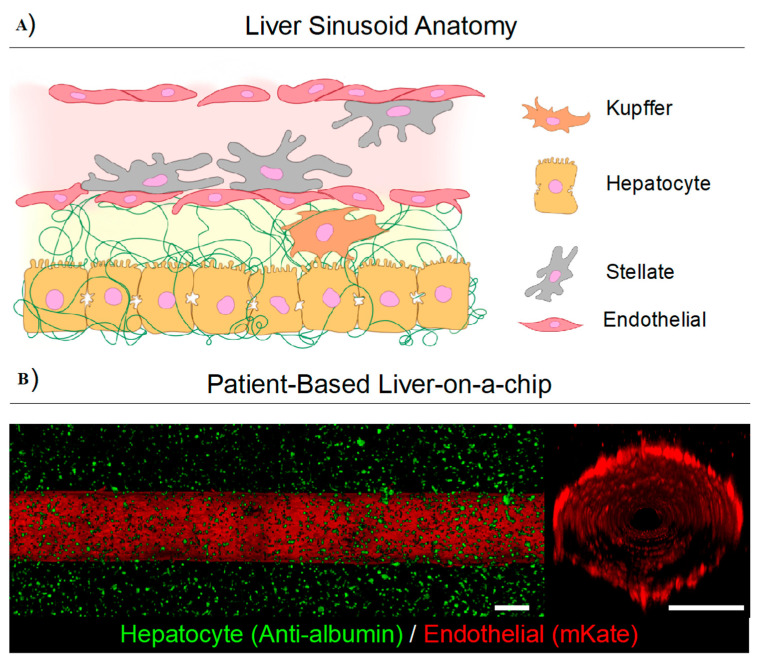
A human liver-on-a-chip device can recapitulate complex cellular architecture of a human liver sinusoid (**A**) by culturing liver-specific cells such as Kupffer, hepatocyte, stellate, and endothelial cells derived from patients within a 3D extracellular matrix (ECM) which emulates the complexity of the liver architecture which would provide a high-throughput system for testing HCC therapies. (**B**) In a preliminary work, Özkan et al. developed a vascularized healthy collagen type 1-based microfluidic in vitro liver platforms and confirmed physiological fidelity based on the measurement of cell and matrix properties and metabolic function. This platform consists of a collagen type 1 ECM tuned to mimic the stiffness of native matrix with healthy hepatocytes (green) surrounding a fully functional blood vessel with endothelial cells (red) lining the lumen. Endothelial cells line the blood vessel, forming a confluent monolayer for measuring transport of biomolecules and chemotherapeutics. Scale bar is 500 µm. Figure 6b reproduced with permission from [85] (Wiley, 2019).

These recent developments in microfluidic platforms and organs-on-a-chip have demonstrated the feasibility of generating natural three-dimensional (3D) models with vascular endothelium under the physiological flow, multicellular architecture, and physiochemical microenvironment of living tissues. Using the patient-based extracellular matrix and cells, microfluidic platforms (Figure 6) have the potential to play a pivotal role as a translational tool for new compounds to enter into the clinic (Özkan et al., 2020, submitted to Lab on a Chip). In addition to drug testing, delivery methods such as embolization with nanoparticles can be examined using these living devices. To recapitulate the complex architecture of liver in vitro, extractive needle methods can be applied for patterning vascular regions including the space of Dissé. Using such methods, Kupffer cells could be cultured with liver sinusoid endothelial cells on the blood vessel and stellate cells could be sandwiched between hepatocytes and cells on the lumen. The inlet and outlet of these devices could be used to deliver new embolization or ablation reagents for optimization studies. The organs-on-a-chip could also be used for further mass spectrometry imaging and analysis to observe ablation region depth and chemical composition variation as a result of the treatments.

However, while collagen is an excellent matrix for promoting physiological cell growth and is the most commonly utilized natural ECM for microfluidic platforms, the optical and thermal properties of collagen-based systems are not representative of human tissue [91]. Furthermore, the melting temperature of rat tail collagen is below the maximum temperature observed during ablative hyperthermic adjunct treatments. In addition, there are concerns about pH stability due to the acidic tumor microenvironment and induced hypoxia from embolization. We have previously developed sodium alginate 3D tumor platforms with optical properties that are very similar to tumor tissue and have utilized them to determine temperature distribution and spatial cell viability following laser therapy [92,93]. 

## 7. Pico-Scale Technology with Mass Spectrometry Imaging: A New Versatile Analytical Method

Concurrent with new systems to mimic tumors with greater fidelity, new analytical technologies will play an increasingly important role in understanding distribution and effects of delivered locoregional therapies. One such emerging technology is mass spectrometry imaging (MSI), which allows for the in situ visualization of the spatial distribution and relative abundance of endogenous and exogenous molecules from tissue sections. Thin sections similar to those used for histopathological evaluation are prepared using appropriate washing/fixation followed by enzymatic digestion and/or matrix application for the class of molecules to be analyzed. Since the first introduction of matrix-assisted laser desorption ionization (MALDI) imaging for proteins in 1997 [94], the methodology has advanced for the analysis of proteins, peptides, lipids, drugs, and metabolites from both fresh frozen and formalin-fixed tissues [95,96]. Commercial MALDI mass spectrometers are capable of imaging at 5–10 µm spatial resolution, nearly at the cellular level. The added molecular information that MSI provides enables a more thorough understanding of disease processes and treatment effects.

To date, MSI has been used to aid in the understanding and molecular diagnosis of numerous diseases including those of the liver. Ščupáková et al. demonstrated lipid changes between steatotic and non-steatotic areas of liver sections along with overall changes in the relative abundance of lipids and metabolites with increasing degree of steatosis [97]. The results of principal component analysis showed dramatic changes between histologically diseased and normally appearing areas. Wattacheril et al. showed that zonal changes occurred in phosphatidylcholine (PC) lipids with progression from normal liver to NAFLD to NASH, with dysregulation and little zonal localization of PC lipids observed in NASH biopsies [98].

More recently, changes in glycan structures have been found to be associated with malignant transformation of the liver [99]. In particular, increases in the complexity of the glycan branching pattern as well as fucosylation were correlated with HCC with over 95% of patients in the study demonstrating elevation of at least one fucosylated glycan structure within the cancerous areas of the biopsy. 

Efficacy of a therapeutic agent is dependent on the successful delivery of the agent to its target tissue. MSI has also shown great utility in the analysis of whether therapeutic agents are delivered to tumors as well as the effects they have on the target and surrounding tissues. In a study of human gastrointestinal stromal tumors and their metastases to liver in the patients who had been treated orally with imatinib, it was found that while imatinib was found to be distributed throughout the primary tumors, no imatinib was observed within the metastatic tumors [100]. Interestingly, imatinib was detected in the adjacent normal liver tissues surrounding the tumors, well above the limit of quantitation, information that would have been lost from LC–MS/MS analysis of homogenized liver biopsies as it is not readily detectable by other means. The lack of imatinib in metastatic tumors helps to explain their resistance to treatment.

MSI has also been employed in the study of a DEB-TACE treatment of a VX2 liver tumor model in rabbits [101]. Drug-eluting beads containing the targeting agent sunitinib were delivered into the artery supplying blood flow to the tumors. MSI was used to evaluate localization and relative abundance of sunitinib and several of its metabolites in and around the area of deposition of the beads at various time points after injection.

**Figure 7:**
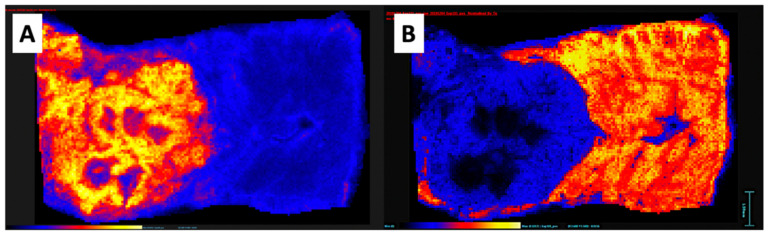
Mass spectrometry images of a single section of rabbit VX2 tumor in the liver acquired by MALDI MSI in the positive ion mode with the α-cyano-4-hydroxycinnamic acid matrix. Color scale blue to yellow, low to high abundance. (**A**) Distribution of the selected analyte (m/z 782.6) This ion/analyte has higher abundance in the tumor. (**B**) Distribution of the selected analyte (m/z 824.6) with higher abundance in the surrounding liver and markedly lower intensity within the tumor.

We have recently started to use MSI for exploration of biomarkers and to map distribution of analytes that could potentially distinguish tumors from healthy tissues using the orthotopic rabbit VX2 tumor model. See an example of distinct features in tumors versus normal tissues in Figure 7. After cryosectioning, the tissues were imaged with MALDI mass spectrometry in the positive ion mode. These images can provide markers for tumor and non-neoplastic tissues while also used to assess damage and to understand better the molecular biology as described previously. 

We have also used MSI to evaluate the effects of thermoembolization in a swine model through the in situ hydrolysis of dichloroacetyl chloride to produce dichloroacetic acid, hydrochloric acid, and heat [102]. Imaging of liver sections collected 24 h after thermoembolization revealed the focal loss of phosphatidylinositol lipids and oleic acid from areas of histological damage, among other changes. Iodide from the ethiodized oil bolus for delivery of dichloroacetyl chloride was detected in the central-most areas of damage and was found to co-localize with other bile salts such as taurodeoxycholate, taurocholic acid, and glycol (cheno)deoxycholate. Given the location and distribution, iodide from the vehicle could potentially represent a surrogate marker for the arterial lumen. Additionally, several lipids were found to be potential markers of ischemia with spatial distribution around the perimeter of the damaged areas of the liver.

## 8. Conclusions

The global burden of HCC is increasing and will continue to do so. Considering the challenges with early diagnosis and the difficulties facing targeted therapies and immune therapies, locoregional therapies are expected to find increased use. Substantial numbers of patients will present for care with large tumor burdens not suited for ablation therapies. Modest success with current transarterial therapies, when viewed through the lens of histopathological correlation, shows that despite progress, there is still much opportunity for improvement. Fortunately, new technologies across the entire workflow of patient care at the micro-, nano-, and pico-scale are on the horizon. High-throughput studies in high-fidelity models are expected to further accelerate our ability to assess these technologies and improve treatment for HCC and other cancers.
